# Exploratory pharmacokinetic-pharmacodynamic characterization and safety of standardized *Andrographis paniculata* aqueous extract capsules in patients with mild COVID-19

**DOI:** 10.3389/fphar.2026.1781740

**Published:** 2026-03-13

**Authors:** Phanit Songvut, Paruspak Payoong, Pilailuk Akkapaiboon Okada, Noppawan Rittapai, Sumitra Suntararuks, Jaratluck Akanimanee, Nuchanart Rangkadilok, Duangchit Panomvana, Porranee Puranajoti, Jutamaad Satayavivad

**Affiliations:** 1 Laboratory of Pharmacology, Chulabhorn Research Institute, Bangkok, Thailand; 2 Center of Excellence on Environmental Health and Toxicology (EHT), OPS, MHESI, Bangkok, Thailand; 3 Department of Medicine, Chulabhorn Hospital, Chulabhorn Royal Academy, Bangkok, Thailand; 4 National Institute of Health, Department of Medical Sciences, Ministry of Public Health, Nonthaburi, Thailand; 5 Translational Research Unit, Chulabhorn Research Institute, Bangkok, Thailand

**Keywords:** *Andrographis paniculata*, COVID-19, pharmacodynamics, pharmacokinetics, safety

## Abstract

**Introduction:**

*Andrographis paniculata* has been used in the management of COVID-19-related conditions during the SARS-CoV-2 pandemic. However, human pharmacokinetic-pharmacodynamic (PK/PD) evidence to support a rationale for safe and evidence-based dosing of standardized extracts in clinical use remains limited. This exploratory study aimed to investigate the pharmacokinetics, preliminary PK/PD relationship, and short-term safety profile of standardized *A. paniculata* aqueous extract capsules in patients with mild COVID-19.

**Methods:**

A single-center PK/PD clinical trial with two sequential phases (single-dose followed by multiple-dose) was conducted. Patients with mild COVID-19 received standardized *A. paniculata* aqueous extract capsules equivalent to 30 mg of andrographolide every 8 h (90 mg/day) for 5 consecutive days, alongside standard symptomatic treatment.

**Results:**

Rapid absorption and elimination with limited systemic exposure supported repeated dosing to maintain exposure over the 5-day period. PK/PD analysis demonstrated a sigmoidal, saturable exposure-response relationship. The mean area under the concentration–time curve (AUC_0-4 h_) on day 5 approached the estimated EAUC_50_ (half-maximal effective exposure) derived from the Emax model. A −2.96 log_10_ reduction in viral load was observed; however, without a placebo control group, it is not possible to conclusively attribute this reduction to the investigational *A. paniculata* aqueous extract capsules. Adverse events were mild, with no hepatotoxicity or renal toxicity observed.

**Conclusion:**

These findings provide preliminary evidence regarding the pharmacokinetics, dose-exposure characteristics, and short-term tolerability of standardized *A. paniculata* aqueous extract capsules, supporting further investigation in patients with mild COVID-19. Larger, placebo-controlled trials are warranted to establish causality and to evaluate clinical benefits.

## Graphical Abstract

**Figure:**
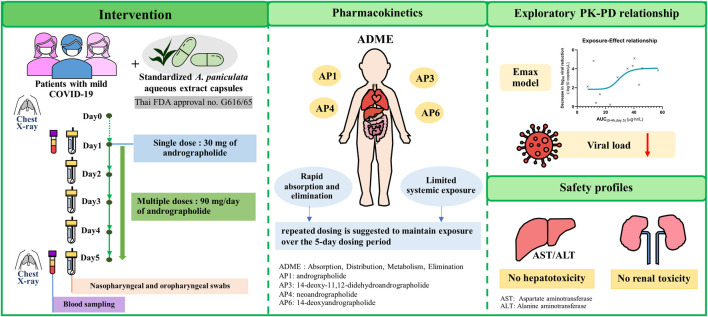

## 1 Introduction

The outbreak of severe acute respiratory syndrome coronavirus 2 (SARS-CoV-2), which caused the coronavirus disease 2019 (COVID-19) pandemic, has posed a global public health challenge (Msemburi et al., 2023), necessitating evidence-based preventive and therapeutic strategies. Although vaccines and antiviral drugs have been recommended for the management of COVID-19, concerns persist regarding their adverse effects and limited efficacy against emerging SARS-CoV-2 variants. Traditional herbal medicines have increasingly been adopted as adjunctive or alternative approaches, particularly for patients with mild to moderate COVID-19 who may benefit from early intervention (Chien et al., 2022; Isidoro et al., 2022).

During the current COVID-19 pandemic, most patients have received SARS-CoV-2 vaccination and typically present with mild symptoms at the early stages of infection. In clinical practice, patients with mild to moderate COVID-19 are generally managed with symptomatic treatment, without the routine use of specific antiviral agents. However, COVID-19 has been associated with greater clinical severity than common influenza (Piroth et al., 2021; Tesch et al., 2024), highlighting the need for adjunctive intervention that may support viral clearance and reduce symptom duration in mild cases. Several studies have investigated *A. paniculata* as an adjunctive intervention in patients with mild to moderate COVID-19 (Intharuksa et al., 2022).

*A. paniculata* (Burm.f.) Nees (family Acanthaceae), a traditional herbal medicine commonly known in Thailand as “Fah Talai Jone”, has increased interest for its potential antiviral activity against SARS-CoV-2 (Zhang et al., 2021; Ratiani et al., 2022; Benjaponpitak et al., 2023). The pharmacological effects of *A. paniculata* are primarily attributed to its diterpene lactone constituents, with andrographolide (AP1) being the major bioactive compound. Other diterpenoids that may contribute to its pharmacological activities include 14-deoxy-11,12-didehydroandrographolide (AP3), neoandrographolide (AP4), and 14-deoxyandrographolide (AP6) (Songvut et al., 2023b).

Recent *in silico* studies have investigated the antiviral mechanisms of andrographolide and its derivatives against SARS-CoV-2, suggesting their potential to interact with multiple viral targets and related pathways involved in SARS-CoV-2 infection. Molecular docking and molecular dynamics simulation analyses have demonstrated strong binding affinities of andrographolide and its derivatives with key SARS-CoV-2 proteins, including 3-chymotrypsin-like protease (3CLpro), papain-like protease (PLpro), spike protein, and RNA-dependent RNA polymerase (RdRp) (Murugan et al., 2021; Khanal et al., 2021). In addition, network pharmacology and pathway enrichment analyses have indicated that the compounds may influence multiple immune- and inflammation-related signaling pathways, including chemokine, MAPK, NF-κB, RAS, p53, HIF-1, cytokine-cytokine receptor interactions, and natural killer cell-mediated cytotoxicity (Khanal et al., 2021).

An *in vitro* study provided additional support for antiviral activity, demonstrating that *A. paniculata* extract and andrographolide exhibited dose-dependent inhibitory effects against SARS-CoV-2 infection in human lung epithelial Calu-3 cells, with IC_50_ values of 0.036 μg/mL and 0.034 μM, respectively (Sa-ngiamsuntorn et al., 2021). Additionally, a recent *in vivo* study using Golden Syrian hamsters infected with the SARS-CoV-2 Delta variant reported that oral administration of *A. paniculata* extract at 1,000 mg/kg/day reduced disease severity, and decreased inflammatory cytokine levels, particularly interleukin-6 (IL-6), in lung tissue compared to the control group (Kongsomros et al., 2024).

For clinical applications, *A. paniculata* has traditionally been used for the treatment of the common cold at a dosage of 20 mg of andrographolide every 8 h, equivalent to a total daily dose of 60 mg of andrographolide for 5 consecutive days (Gabrielian et al., 2002). However, a retrospective cohort study evaluated the use of a higher oral dosage of andrographolide (180 mg/day) in patients with COVID-19 and reported potential benefits, particularly in patients with mild symptoms or asymptomatic infection. Notably, a reduced incidence of pneumonia was observed among non-vaccinated patients during the early stages of COVID-19 (Benjaponpitak et al., 2023). Despite the clinical use of high-dose andrographolide (180 mg/day) in mild to moderate COVID-19, the safety concerns still remain. A previous study (Songvut et al., 2023b) evaluated the safety and pharmacokinetics of *A. paniculata* aqueous extract capsules administered at this dosage (180 mg/day) for 7 days in healthy volunteers reported mild and reversible in liver enzymes. Specifically, increases in aspartate aminotransferase (AST) and alanine aminotransferase (ALT) levels were observed in 4 out of 12 participants (Songvut et al., 2023b). Furthermore, pharmacokinetic studies have demonstrated that both aqueous and ethanolic extracts of *A. paniculata* exhibited poor oral bioavailability, with the aqueous extracts demonstrating relatively greater absorption than ethanolic extracts (Songvut et al., 2023b; Songvut et al., 2025). In addition, ethanolic extract has been reported to exhibit non-linear pharmacokinetics, in which plasma andrographolide concentrations do not increase proportionally with increasing administered doses (Songvut et al., 2025), thereby necessitating careful dose selection and exposure-based dose adjustment.

While *A. paniculata* ethanolic extract has been used in clinical studies for the treatment of COVID-19 and upper respiratory tract infections, available evidence indicates that the aqueous extract exhibits significantly higher dissolution (Rangkadilok et al., 2024) and greater systemic exposure (Songvut et al., 2023b) compared to ethanolic extract (Rangkadilok et al., 2024; Songvut et al., 2025). Based on available evidence suggesting higher dissolution and systemic exposure of aqueous extracts relative to ethanolic extracts, an aqueous extract formulation was selected for the present exploratory study. Nevertheless, the critical gap remains regarding the PK/PD characteristics and overall safety profile of *A. paniculata* extracts in patients with COVID-19. Accordingly, this exploratory clinical pharmacology study was designed to fill this critical knowledge gap by characterizing the human pharmacokinetics of standardized *A. paniculata* aqueous extract capsules, evaluating preliminary exposure-response relationships, and examining short-term safety in patients with mild COVID-19.

## 2 Materials and methods

### 2.1 Chemicals

The analytical standards for the four target compounds were purchased from Phytolab GmbH & Co.KG (Vestenbergsgreuth, Germany), including AP1 (99.97% purity), AP3 (100.00% purity), AP4 (99.59% purity), and AP6 (96.87% purity). The internal standard digoxin (98.3% purity) was purchased from Merck (Darmstadt, Germany). High-performance liquid chromatography (HPLC)-grade acetonitrile and methanol were supplied by Merck (Darmstadt, Germany). Ultra-pure water used in the Liquid Chromatography-Tandem Mass Spectrometry (LC-MS/MS) analysis was obtained from a Milli-Q purification system (Millipore, Bedford, MA, USA).

### 2.2 Study medication

The investigational product used in this study was a commercially available standardized *A. paniculata* aqueous extract capsule prepared from aerial parts of the plant (Brand PC-1999®, Lot No. 119010921). Each capsule contained 230 mg of *A. paniculata* aqueous extract, equivalent to 10 mg of andrographolide as the active pharmaceutical ingredient. The product was manufactured by Panaosod Co., Ltd. (Thailand) in compliance with Good Manufacturing Practice under the Pharmaceutical Inspection Co-operation Scheme (GMP PIC/S) and was registered with the Thai Food and Drug Administration (Thai FDA, Ministry of Public Health) under registration number G616/65. Quality control testing was conducted in accordance with the Thai Herbal Pharmacopoeia (Department of Medical Sciences, Ministry of Public Health, 2021), including assessments of loss on drying, weight variation, disintegration, and microbial contamination limits. The contents of andrographolide and other diterpenoids were analytically determined prior to clinical use. The results are summarized in Table 1, showing an andrographolide content of 10.28 mg per capsule. Quantitative determination of the four diterpenoids in the standardized *A. paniculata* aqueous extract capsules (Table 1) was performed using high-performance liquid chromatography with diode-array detector (HPLC-DAD), following a previously published analytical method (Pholphana et al., 2013). Representative HPLC chromatograms are provided in the Supplementary Figure S1 and the chemical structures of the four major active diterpenoids (AP1, AP3, AP4, and AP6) are presented in Supplementary Figure S2.

**TABLE 1:** The content of four diterpenoids in standardized *A. paniculata* aqueous extract capsules.

Compounds	Active diterpenoid contents mean ± SD (mg/capsule, n = 3)
AP1: andrographolide	10.28 ± 0.03
AP3: 14-deoxy-11, 12-didehydroandrographolide	2.31 ± 0.03
AP4: neoandrographolide	2.97 ± 0.05
AP6: 14-deoxyandrographolide	2.68 ± 0.03
Average net weight of content (mean ± SD, n = 20)	352.54 ± 10.30 (mg/capsule)

For clinical dosing, the administered dose was defined based on the label amount, which required the andrographolide content (10 mg per capsule) in accordance with Thai FDA quality control requirements. Accordingly, participants received three capsules per dose to achieve a nominal dose equivalent to 30 mg of andrographolide per administration, based on the labeled content, resulting in a total daily dose of 90 mg of andrographolide.

### 2.3 Pharmacokinetics and pharmacodynamics (PK/PD) clinical trial

#### Ethics statement

The clinical study was conducted in accordance with the principles of the Declaration of Helsinki and Good Clinical Practice (GCP) guidelines. The study protocol received ethical approval from the Institutional Review Board (IRB) of the Chulabhorn Research Institute (Protocol No. 003/2566, Date: 29 September 2023). The study was registered in the Thai Clinical Trials Registry (TCTR) with registration number TCTR20241123001, Date: 23 November 2024, (URL: https://www.thaiclinicaltrials.org/show/TCTR20241123001) following the WHO International Clinical Trials Registry Platform (WHO-ICTRP).

Prior to enrollment, all participants provided written informed consent after receiving comprehensive information regarding the study’s purpose, procedures, potential risks and benefits, and their rights as research volunteers. The informed consent process was conducted in the participants’ native language, with sufficient time provided for questions and thoughtful consideration before consent was obtained. Participant confidentiality and data privacy were maintained throughout the study period.

#### 2.3.1 Study participants and enrollment

##### 2.3.1.1 Participant population

This study enrolled 12 adult patients diagnosed with COVID-19 who met the inclusion and exclusion criteria. Participants were recruited through screening of eligible COVID-19 patients.

##### 2.3.1.2 Inclusion criteria

Participants were eligible for enrollment if they met all of the following criteria: (1) Thai nationality, age 18–60 years; (2) Patients were classified as having mild disease according to the World Health Organization (WHO) Clinical Management Guidelines for COVID-19 (World Health Organization, 2020): (2.1) Laboratory-confirmed SARS-CoV-2 infection by RT-PCR within 72 h of symptom onset; (2.2) Symptomatic infection with clinical signs of COVID-19 (e.g., fever, cough, sore throat, malaise, fatigue) without evidence of viral pneumonia or hypoxia; (2.3) No signs of severe disease including dyspnea, severe respiratory distress, or requirement for supplemental oxygen; and (3) willingness to participate voluntarily in this study.

##### 2.3.1.3 Exclusion criteria

Participants were excluded from the study if they presented with any of the following conditions: (1) abnormal findings on chest X-ray suggestive of pulmonary complications; (2) clinical symptoms consistent with pneumonia or blood oxygen saturation (SpO_2_) below 95% at rest; (3) current or recent use of antiviral medications including molnupiravir, favipiravir, remdesivir, lopinavir/ritonavir, interferon-β1a, chloroquine, hydroxychloroquine or other antiviral medications; (4) history of allergic reactions to *A. paniculata* or andrographolide; (5) pregnant women, breastfeeding mothers, or women planning to become pregnant during the study period; (6) current enrollment in other COVID-19 treatment studies or clinical trials; (7) administration of herbal medicines or products containing *A. paniculata* extract or andrographolide within 5 days prior to enrollment; (8) smoking exceeding 10 cigarettes per day; (9) hypertensive patients (blood pressure >140/90 mmHg) receiving antihypertensive medication; (10) history of hepatic disease, cardiac conditions, rheumatic heart disease, or nephropathy secondary to previous group A *Streptococcus* infection; (11) current use of anticoagulant medications including clopidogrel, warfarin, or aspirin; (12) high-risk conditions for severe COVID-19 progression were excluded: (12.1) chronic obstructive pulmonary disease (COPD) or other chronic respiratory conditions such as advanced tuberculosis, bacterial pneumonia, or poorly controlled asthma; (12.2) chronic kidney disease, acute kidney injury, or end-stage renal disease; (12.3) cardiovascular diseases, including congenital heart conditions; (12.4) cerebrovascular disease; (12.5) uncontrolled diabetes mellitus; (12.6) obesity with body mass index (BMI) >35 kg/m^2^; (12.7) hepatic cirrhosis or active hepatitis; (12.8) immunocompromised states, including HIV infection; (12.9) lymphopenia with absolute lymphocyte count <1,000 cells/μL; or (13) any condition determined by the investigating physician to potentially increase risk or adversely affect the safety of the participant during the study period.

##### 2.3.1.4 Discontinuation and subject withdrawal criteria

Participants were discontinued and withdrawn from this study under the following: (1) continuation with the study would be harmful to the participant’s wellbeing as determined by the clinical investigator; (2) participants who developed severe or critical COVID-19 symptoms during the study period, including dyspnea requiring assisted ventilation, respiratory failure with inability to breathe spontaneously, pulmonary parenchymal damage evident on chest radiography, or other life-threatening complications necessitating intensive care management; (3) initiation of concurrent antiviral treatment including molnupiravir, favipiravir, remdesivir, lopinavir/ritonavir, interferon-β1a, chloroquine, hydroxychloroquine, or other antiviral medications during the study period; (4) occurrence of serious adverse events (SAEs), specifically severe adverse drug reactions posing life-threatening risks or other severe adverse effects significantly compromising participant safety and wellbeing; (5) voluntary withdrawal by the participant’s own request without requirement for justification; or (6) termination of the entire study by the funding sponsor for any reason including participant safety concerns or regulatory requirements.

#### 2.3.2 Study design and interventions

**FIGURE 1:**
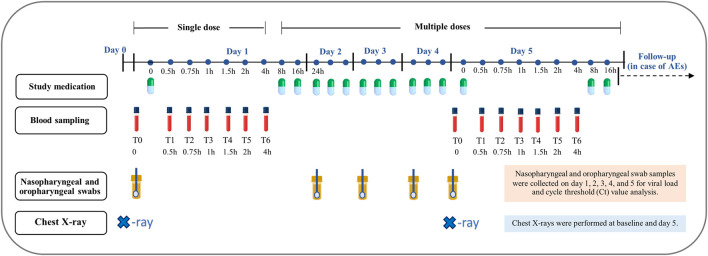
Study outline and sampling schedule.

This study was an open-label, single-center, pharmacokinetic, pharmacodynamic, and safety study of standardized *A. paniculata* aqueous extract capsules in patients with COVID-19. The study design consisted of two sequential phases: single-dose pharmacokinetic assessment followed by multiple-dose administration with safety monitoring (Figure 1). Participants received symptomatic treatment, including paracetamol, antihistamine and antitussive agents, which were administered on an as-needed basis (PRN). The clinical study was conducted at the acute respiratory infection (ARI) clinic, medical center, Chulabhorn Royal Academy, Chulabhorn Hospital, Bangkok, Thailand.

##### 2.3.2.1 Baseline and single-dose investigation

Enrolled participants underwent baseline assessments on day 0, including vital signs monitoring, blood oxygen saturation (SpO_2_) monitoring, chest X-ray examination, and collection of nasopharyngeal and oropharyngeal swab samples. An overnight fasting period of at least 8 h was required for all participants before receiving the study medication. On day 1, eligible participants with confirmed COVID-19 diagnosis received a single oral dose of standardized *A. paniculata* aqueous extract capsules equivalent to 30 mg of andrographolide, administered with 240 ± 2 mL of drinking water. Participants were required to maintain an upright sitting position for 1-h post-dosing, with water intake restricted during this period. For pharmacokinetic analysis, serial blood samples (6 mL each) were collected via forearm venous cannulation at following time points: pre-dose (0 h), and at 0.5, 0.75, 1, 1.5, 2, and 4 h post-administration. Additional blood samples (11 mL) were collected at pre-dose for safety laboratory assessments. Nasopharyngeal and oropharyngeal swab samples were collected on days 1, 2, 3, 4, and 5 for RT-PCR and viral load analysis. A standard meal was provided after the 4-hour blood sample collection.

The 0–4 h sampling window was based on prior pharmacokinetic data following oral administration of *A. paniculata* aqueous extract capsules in healthy volunteers, which demonstrated that andrographolide decreased to below the lower limit of quantification by approximately 4 h post-dose (Songvut et al., 2023b). Moreover, because the present study involved patients with active COVID-19, ethical and infection control considerations necessitated minimizing on-site duration. Accordingly, the 0–4 h sampling window was selected to explore the absorption phase, peak plasma concentration, and early systemic exposure within the context of this exploratory investigation.

##### 2.3.2.2 Sequential single- and multiple-dose study

After administration of the first dose, intensive blood sampling was performed over the initial 4-hour period to characterize the single-dose pharmacokinetic profile. The pharmacokinetic parameters derived from this 0–4 h window therefore represent exposure following a single dose prior to accumulation.

Upon completion of the 4-hour single-dose pharmacokinetic assessment, participants continued the dosing schedule on the same day. The second and third doses were administered at 8 and 16 h after the first dose, respectively, thereby initiating the multiple-dose phase on day 1 following completion of the single-dose pharmacokinetic evaluation. As illustrated in Figure 1, a total of three doses were administered on day 1. Dosing was then continued three times daily at 8-hour intervals through day 5, providing a total daily dose equivalent to 90 mg/day of andrographolide (30 mg per dose, three doses per day). On day 5, participants returned to the clinical site for multiple-dose pharmacokinetic investigation after an overnight fast of at least 8 h. Pre-dose assessments included vital signs, SpO_2_ measurement, and chest X-ray. Additionally, nasopharyngeal and oropharyngeal swab samples were obtained for RT-PCR and viral load analysis. Multiple-dose pharmacokinetic blood sampling followed the same protocol as the single-dose phase.

The 8-hour dosing interval was selected based on the standard clinical use of *A. paniculata* capsules in Thailand during the COVID-19 pandemic and existing product labeling. In a prior study conducted in healthy volunteers using standardized *A. paniculata* aqueous extract capsules, administration at 8-hour intervals demonstrated an acceptable safety and tolerability profile (Songvut et al., 2023b). As the present investigation represents the first pharmacokinetic study of this formulation in patients with mild COVID-19, the 8-hour dosing schedule was aligned with the regimen previously shown to be safe and well tolerated in healthy subjects. Although plasma andrographolide concentrations declined to below the lower limit of quantification after 4 h post-dose, this may not reflect the absence of pharmacological activity throughout the remaining dosing interval. Andrographolide has been widely reported to exert multitarget pharmacological actions, including modulation of inflammatory cytokine production (Lim et al., 2012; Dai et al., 2019). Through these mechanisms, its biological effects may persist beyond the period of quantifiable plasma exposure.

#### 2.3.3 Safety monitoring and tolerability assessments

Safety and tolerability were assessed through the monitoring of adverse events (AEs), vital signs, and clinical laboratory parameters throughout the study period. Participant-reported adverse events were graded according to severity (mild, moderate, or severe) and classified based on their relationship to the investigational product as definitely related, probably related, possibly related, unlikely related, or unrelated. Safety clinical laboratory assessments were conducted at pre-dose (day 1) and post-multiple dosing (day 5), involving collection of 11 mL blood samples for analysis of complete blood count (white blood cell, neutrophil, lymphocyte, monocyte, eosinophil, basophil, hemoglobin, hematocrit, red blood cell, and platelet), fasting blood glucose, blood urea nitrogen, creatinine, estimated glomerular filtration rate (eGFR), total bilirubin, albumin, uric acid, AST, ALT, C-reactive protein (CRP), interleukin-6 (IL-6), total cholesterol, high-density lipoprotein (HDL) cholesterol, low-density lipoprotein (LDL) cholesterol, and triglycerides. Vital signs monitoring and SpO_2_ measurements were performed throughout the study period to assess participant safety and clinical status. All participants were monitored for adverse events from enrollment through study completion.

Clinical efficacy endpoints were not evaluated in this study. The investigation focused on pharmacokinetic characterization, exploratory exposure-response analysis, and short-term safety assessment. Thus, symptom duration, time to symptom resolution, disease progression, and quality-of-life outcomes were not systematically assessed.

#### 2.3.4 Sample preparation for LC-MS/MS analysis

Blood samples collected for pharmacokinetic analysis were processed immediately according to the previously published validated method (Songvut et al., 2023a). Samples were centrifuged at 3,200 × *g* at 4 °C for 10 min to obtain plasma. Protein precipitation extraction was performed as previously described. Briefly, 100 μL of plasma was transferred to a microtube and combined with 400 μL of methanol containing digoxin internal standard (2 ng/mL). Following thorough vortex mixing for 5 min, samples were centrifuged at 18,800 × *g* for 10 min at 4 °C. The clear supernatant was filtered through a 0.22 μm PVDF syringe filter (Chrome Tech, Inc., USA) and collected in amber vials, then stored at 4 °C in LC autosampler until LC-MS/MS analysis on the same day as sample collection and extraction.

#### 2.3.5 LC-MS/MS bioanalytical method for plasma concentration analysis

Plasma concentrations of the four target bioactive compounds, including AP1, AP3, AP4, and AP6, were determined using LC-MS/MS at the Laboratory of Pharmacology, Chulabhorn Research Institute, Bangkok, Thailand. The bioanalytical method used in this study was modified and partially validated based on our previously published and fully validated method (Songvut et al., 2023a).

Chromatographic separation was performed using a VertiSep AQS C18 analytical column (100 × 3.0 mm, 3 μm particle size; Vertical Chromatography Co., Ltd., Thailand) equipped with a compatible guard cartridge (Fusion-RP, 4.0 × 2.0 mm; Phenomenex Co., Ltd, USA). The mobile phase system comprised (A) ultrapure water and (B) acetonitrile with the following gradient program: 45% B (0–1.5 min), linear increase to 90% B (1.5–3.0 min), 90% B hold (3.0–5.0 min), 90% B to 45% B (5.0–5.1 min), followed by re-equilibration to 45% B (5.1–8.5 min). Analysis was conducted with a 10 μL injection volume, 0.5 mL/min flow rate, and column temperature maintained at 40 °C.

The LC-MS/MS system (Shimadzu, Kyoto, Japan) consisted of an LC-40D XR pump unit with a SIL-40C XR autosampler coupled to an LCMS-8060NX triple quadrupole mass spectrometer equipped with an electrospray ionization (ESI) interface. The instrument was operated using multiple-reaction monitoring (MRM) detection in negative electrospray ionization mode for quantitative analysis. System control and data processing were performed using LabSolutions software (Shimadzu, Kyoto, Japan). Quantitative analysis was based on specific MRM transitions (precursor → product ion) for andrographolide (349.15 → 287.30), 14-deoxy-11,12-didehydroandrographolide (331.2 → 239.2), neoandrographolide (479.25 → 317.30), 14-deoxyandrographolide (333.20 → 285.35), and internal standard digoxin (779.45 → 649.40). MS chromatogram and retention time are provided in the Supplementary Figure S3. The MS conditions were as follows: nebulizer gas flow = 3 L/min, heating gas flow = 15 L/min, interface temperature = 250 °C, DL temperature = 250 °C, heat block temperature = 400 °C, drying gas flow = 18 L/min.

The bioanalytical method validation demonstrated good selectivity, with no interference from endogenous plasma components observed at the retention times of any of the analytes. The lower limit of quantification (LLOQ) was 4.69, 2.34, 1.88, and 3.75 ng/mL for AP1, AP3, AP4, and AP6, respectively. Method accuracy and precision were within acceptable limits, defined as ±20% at the LLOQ and ±15% at low-, medium-, and high-quality control (LQC, MQC, and HQC) concentrations. At the LLOQ level, the accuracy ranged from 80.86%–112.31%, 82.98%–114.42%, 82.25%–116.38%, and 81.84%–119.89%, while the precision, expressed as %CV, ranged from 8.28%–13.53%, 5.92%–8.10%, 3.19%–14.74%, and 6.77%–14.51% for AP1, AP3, AP4, and AP6, respectively. The overall QC accuracy (%) at the LQC, MQC, and HQC levels ranged from 85.13%–114.81%, 85.12%–114.96%, 86.19%–114.92%, and 85.40%–113.81%, while the overall QC precision (%CV) ranged from 2.94%–11.66%, 1.18%–9.27%, 3.19%–9.29%, and 3.45%–11.67% for AP1, AP3, AP4, and AP6, respectively.

#### 2.3.6 Pharmacokinetic analysis

Analysis of pharmacokinetic parameters following single and multiple oral dosing was performed using PK Solutions software version 2.0 (Summit Research Services). A non-compartmental analysis was performed to calculate all relevant pharmacokinetic variables. Plasma concentration-time profiles for both single and repeated dose administrations were constructed using GraphPad Prism version 10.2.1 (GraphPad Software, USA). Peak plasma concentrations (Cmax) and time to reach peak plasma concentration (Tmax) for each compound were determined by direct observation from the generated plasma concentration-time profiles. The area under the concentration-time curve (AUC) from administration to the final measurable time point (AUC_0-t_) was computed using the linear trapezoidal method. Extrapolation to infinity ( ${\text{AUC}}_{0-\infty }$ ) was performed using the relationship ${\text{AUC}}_{0-\infty }$ = AUC_0-t_ + (C_last_/k_el_), where k_el_ represents the terminal elimination rate constant derived from log-linear regression analysis of the elimination phase, and C_last_ is the final quantifiable plasma concentration. The apparent volume of distribution (Vd/F) following oral administration was calculated using the equation Vd/F = Dose/(AUC × k_el_), where F represents oral bioavailability. Apparent oral clearance (Cl/F) was determined as Cl/F = Dose/AUC. The terminal elimination half-life (t_1/2_) was calculated using the relationship t_1/2_ = ιn_2_/k_el_, where ιn_2_ = 0.693. The mean residence time (MRT) represents the average duration that drug molecules remain within the body following administration.

#### 2.3.7 Reverse-transcription droplet digital PCR (RT-ddPCR) assay for RdRp viral load

RT-ddPCR was performed using primers and probes targeting the RdRp gene, a sensitive and specific marker for SARS-CoV-2 detection, based on the RT-PCR method developed by the National Institute of Health of Thailand (Thai NIH), an accredited reference laboratory of the Department of Medical Sciences, Ministry of Public Health. The assay was conducted in combination with the One-Step RT-ddPCR Advanced Kit for Probes (Bio-Rad Laboratories Inc., Hercules, CA, USA). The reaction mixture (20 µL) contained reverse transcriptase and DNA polymerase enzymes in a buffer composed of standard RT-PCR components and RdRp primers/probe. After adding 5 μL of RNA template, the mixture was partitioned into water-in-oil emulsion using a droplet generator (QX200™ AutoDG Droplet Digital PCR System, Bio-Rad Laboratories Inc., Hercules, CA, USA) to generate approximately 1-nL droplets. In each experiment, reagent controls were included by substituting purified water for RNA samples.

Amplification was performed in a thermal cycler (T-100™, Bio-Rad Laboratories Inc., Hercules, CA, USA) under the following conditions: 45 °C for 60 min for cDNA synthesis, followed by 95 °C for 10 min to inactivate reverse transcriptase and activate DNA polymerase. Subsequently, 40 cycles of RdRp gene amplification were performed at 95 °C for 15 s and 50 °C for 1 min. The remaining enzymes were then inactivated and droplet stabilization was achieved at 98 °C for 10 min, followed by 4 °C for at least 10 min. Fluorescence signals were detected in the FAM channel using a droplet reader (QX200™ Droplet Reader, Bio-Rad Laboratories Inc., Hercules, CA, USA).

Data analysis was performed using QuantaSoft™ Software v.1.7.4.0917 (Bio-Rad Laboratories Inc., Hercules, CA, USA). The number of positive droplets and the total droplet count were used to calculate the concentration in copies/μL of reaction, together with the 95% confidence interval (95% CI). Multiplying this value by the total reaction volume (20 μL) yielded the result in copies/reaction, which corresponded to the number of copies present in the 5 μL RNA sample.

#### 2.3.8 Real-time RT-PCR preparation and procedure for RdRp Ct value analysis

Real-time RT-PCR was performed using primers and probes targeting the RdRp gene of SARS-CoV-2, following the protocol developed by the National Institute of Health, Department of Medical Sciences, Thailand. The master mix was prepared to a total volume of 20 µL per reaction, comprising the following components: RdRP-F and RdRP-R primers at a final concentration of 0.8 µM each, RdRP probe at a final concentration of 0.2 µM (total volume 2.4 µL), CAPITAL qPCR Probe Mix (4x) at 5 μL, RTase with RNase Inhibitor (20x) at 1 μL, and Enhancer at 5.3 µL. Subsequently, 5 µL of extracted RNA template was added to each reaction.

The amplification was performed using a Bio-Rad CFX96 Real-Time PCR Detection System (Bio-Rad, USA) with the following thermal cycling conditions: Step 1, reverse transcription at 50 °C for 30 min; Step 2, pre-incubation at 95 °C for 2 min (1 cycle); and Step 3, amplification consisting of denaturation at 95 °C for 15 s followed by annealing/extension at 55 °C for 45 s, repeated for 45 cycles. Results were evaluated based on the amplification curve morphology and Ct values. Samples were considered positive (detected) for the RdRp gene if an S-shaped amplification curve was observed with a Ct value ≤40. Samples without fluorescent signals or with Ct values exceeding the established threshold were reported as negative (not detected).

#### 2.3.9 Assay for ORF1ab Ct value

The ORF1ab gene is generally used in routine diagnostic testing owing to its high sensitivity, whereas RdRp is often used for research purposes due to its greater specificity (Vogels et al., 2020). In the present study, the RdRp gene was used as the primary target for viral load assessment and Ct value interpretation. Additionally, the ORF1ab gene was analyzed to confirm the RdRp Ct values.

Viral RNA detection and quantification for ORF1ab Ct value determination were performed using the cobas SARS-CoV-2 assay on a cobas 6800 system (Roche Diagnostics, Mannheim, Germany) at the Central Laboratory Facility, Chulabhorn Hospital, Bangkok, Thailand. The automated platform integrated sample preparation, nucleic acid extraction, PCR amplification, and real-time detection with computerized data management. Analysis was performed using primers targeting ORF1ab region, a highly sensitive and specific viral target for SARS-CoV-2 detection. During extraction, nucleic acids from each specimen and the RNA internal control (IC) were processed simultaneously. Briefly, samples were lysed using proteinase and lysis buffer at elevated temperature to release viral RNA. After washing to remove proteins, cellular debris, and PCR inhibitors, nucleic acids were eluted at elevated temperature and transferred into the amplification mixture.

### 2.4 Statistical analysis

Statistical analysis was performed using IBM SPSS Statistics version 22.0 (IBM Corp., Armonk, NY, USA). The normality of data distribution was assessed using the Shapiro–Wilk test. For pharmacokinetics and clinical safety laboratory parameters, continuous variables with normal distribution were presented as the mean ± standard deviation (SD), whereas non-normally distributed variables, including Tmax and t_1/2_, were reported as the median and interquartile range (IQR). Comparative analyses of pharmacokinetic and clinical laboratory parameters between single- and multiple-dose regimens, as well as between pre- and post-oral dosing, were conducted using paired t-tests for normally distributed data or Wilcoxon signed-rank tests for non-parametric data, as appropriate. A *p-*value of less than 0.05 was considered statistically significant. Graphical representations were generated using GraphPad Prism version 9.3.0 (GraphPad Software, San Diego, CA, USA).

Viral load analyses were conducted for descriptive and exploratory purposes and were not intended to provide confirmatory evidence of antiviral efficacy. Viral load was expressed as the geometric mean (GM) with geometric standard deviation (GSD), and log_10_-transformed values were used for statistical comparisons. Ct values from RT-PCR were presented as mean ± SD. To investigate the PK/PD relationship of andrographolide, a standard exposure-effect model was applied to the observed AUC values (Chawla et al., 2025) and the corresponding log_10_ viral load reduction (day 5). The relationship was calculated using GraphPad Prism version 9.3.0 (GraphPad Software, San Diego, CA, USA) by the following equation:

$$\text{Effect}=\text{Emax}\times {\text{AUC}}^{\gamma }\;/\;\left({\text{EAUC}}_{50}^{\gamma }+{\text{AUC}}^{\gamma }\right),$$

where effect represents the log_10_ viral load reduction, Emax is the maximum model-estimated achievable effect, AUC is the andrographolide area under the concentration-time curve from 0 to 4 h (on day 5), EAUC_50_ is the AUC value producing 50% of the maximum effect estimated by the PK/PD model, and ^γ^ is the Hill coefficient determining the sigmoidicity of the concentration-effect relationship. Nonlinear regression was performed without weighting. There were no missing data for the variables included in the analysis.

Sample size determination followed a general guide for sample size estimation in clinical trials (Sakpal, 2010). Based on previous pharmacokinetic data (n = 12/group) for *A. paniculata* aqueous extract capsules (Songvut et al., 2023b), the required sample size was calculated assuming 80% statistical power and a two-sided significance level of *α* = 0.05.

## 3 Results

### 3.1 Participant characteristics and CONSORT diagram

**FIGURE 2:**
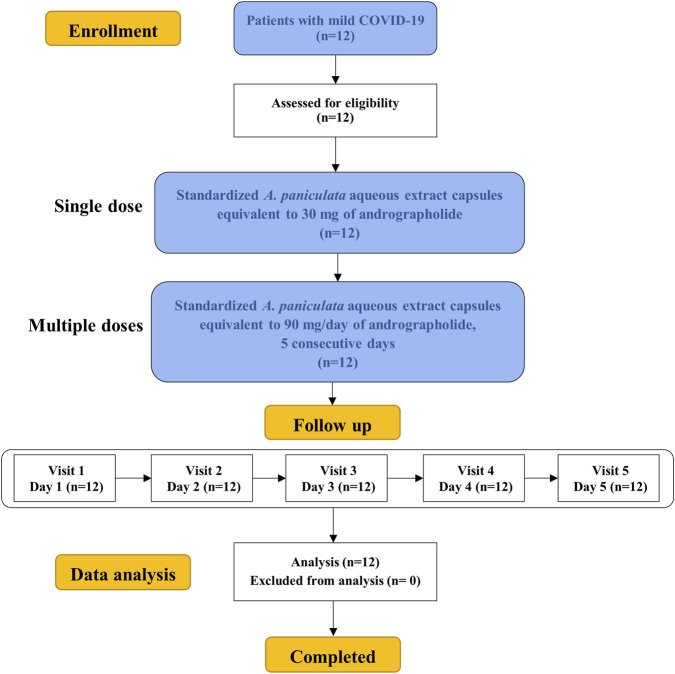
CONSORT flow diagram.

Figure 2 CONSORT flow diagram, A total of 12 patients with mild COVID-19 were enrolled and assessed for eligibility in this study. All participants met the inclusion criteria and were assigned to receive standardized *A. paniculata* aqueous extract capsules equivalent to 30 mg of andrographolide as a single oral dose on day 1. The same participants subsequently received a multiple-dose regimen of andrographolide at 90 mg/day, administered as 30 mg every 8 h for 5 consecutive days. All participants completed the study protocol and attended all scheduled follow-up visits from day 1 through day 5. There were no dropouts, withdrawals, or protocol deviations. No adverse events leading to discontinuation were reported, and compliance was 100%. Consequently, data from all 12 participants were included in the final analysis of PK/PD and clinical safety outcomes. All participants had received their latest vaccine dose more than 1 year before enrollment.

### 3.2 Pharmacokinetics

#### 3.2.1 Comparative pharmacokinetic parameters

**FIGURE 3:**
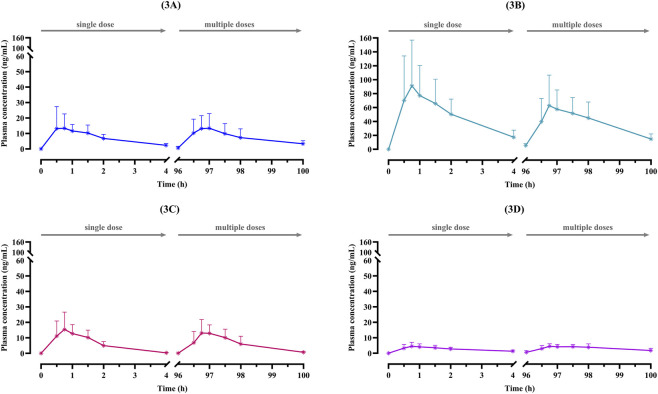
Mean plasma concentration-time profiles of four diterpenoids; **(A)** AP1: andrographolide; **(B)** AP3: 14-deoxy-11, 12-didehydroandrographolide; **(C)** AP4: neoandrographolide; and **(D)** AP6: 14-deoxyandrographolide, after single (30 mg of andrographolide) and multiple (90 mg/day of andrographolide) oral administration of standardized *A. paniculata* aqueous extract capsules for 5 consecutive days in patients with mild COVID-19. Data are presented as the mean ± SD (n = 12).

The four major diterpenoids (AP1, AP3, AP4, and AP6) were rapidly absorbed, with peak plasma concentrations achieved within the first hour following both single- and multiple-dose administration (Figure 3; Table 2). Systemic exposure differed among the four diterpenoids, with AP3 demonstrating the highest AUC_0-4h_ and ${\text{AUC}}_{0-\infty }$ , followed sequentially by AP1, AP4, and AP6. Differences in Vd/F were also observed, with AP1 exhibiting extensive tissue distribution, whereas AP3 exhibited more limited distribution relative to AP1, AP4, and AP6. Considering elimination, AP1 exhibited the highest clearance values, whereas AP3 showed the lowest, resulting in greater plasma concentrations of AP3 compared with the other diterpenoids. Furthermore, elimination kinetics varied substantially among the compounds. AP4 showed the shortest t_1/2_, while AP1, AP3 and AP6 demonstrated similar t_1/2_ values. Notably, AP6 exhibited relatively low plasma concentrations and more rapid clearance, suggesting a limited potential for systemic accumulation.

**TABLE 2:** Pharmacokinetic parameters following single- and multiple-dose oral administration of standardized *A. paniculata* aqueous extract capsules for 5 consecutive days in patients with mild COVID-19.

PK parameters	30 mg andrographolide (n=12)	30 mg andrographolide × 3 times 90 mg/day (n=12)
Andrographolide (AP1)		
Cmax (µg/L)^a^	18.63 ± 11.81	17.82 ± 10.56
Tmax (h)^b^	0.75 [0.31]	0.75 [0.5]
AUC_0-4h_ (µg ⋅ hr/L)^a^	28.54 ± 8.72	30.12 ± 15.83
AUC_0-inf_ (µg ⋅ hr/L)^a^	32.81 ± 8.91	N/A
MRT (h)^a^	2.15 ± 0.69	4.19 ± 3.88
Vd/F (L/Kg)^a^	27.47 ± 13.55	41.02 ± 32.22
Cl/F (L/h/Kg)^a^	14.48 ± 3.66	15.05 ± 11.67
Half life (h)^b^	1.21 [0.19]	1.48 [1.99]
14-deoxy-11,12-didehydroandrographolide (AP3)		
Cmax (µg/L)^a^	105.63 ± 60.88	81.78 ± 41.63
Tmax (h)^b^	0.75 [0.75]	1.00 [0.75]
AUC_0-4h_ (µg ⋅ hr/L)^a^	191.31 ± 84.24	150.47 ± 50.52
AUC_0-inf_ (µg ⋅ hr/L)^a^	231.91 ± 101.94	N/A
MRT (h)^a^	2.37 ± 1.08	2.36 ± 0.63
Vd/F (L/Kg)^a^	1.80 ± 0.85	2.17 ± 0.84
Cl/F (L/h/Kg)^a^	0.96 ± 0.42	1.15 ± 0.40
Half life (h)^b^	1.22 [0.30]	1.21 [0.33]
Neoandrographolide (AP4)		
Cmax (µg/L)^a^	19.56 ± 9.51	18.15 ± 6.58
Tmax (h)^b^	0.75 [0.25]	1.00 [0.38]
AUC_0-4h_ (µg ⋅ hr/L)^a^	24.54 ± 6.71	24.04 ± 7.87
AUC_0-inf_ (µg ⋅ hr/L)^a^	24.81 ± 6.72	N/A
MRT (h)^a^	1.33 ± 0.29	1.46 ± 0.32
Vd/F (L/Kg)^a^	4.39 ± 2.18	6.38 ± 3.61
Cl/F (L/h/Kg)^a^	6.90 ± 2.03	7.02 ± 1.97
Half life (h)^b^	0.52 [0.25]	0.58 [0.09]
14-Deoxyandrographolide (AP6)		
Cmax (µg/L)^a^	5.45 ± 2.20	5.81 ± 1.63
Tmax (h)^b^	0.88 [0.38]	0.75 [0.88]
AUC_0-4h_ (µg ⋅ hr/L)^a^	10.31 ± 3.62	12.53 ± 5.00
AUC_0-inf_ (µg ⋅ hr/L)^a^	14.04 ± 8.25	N/A
MRT (h)^a^	2.76 ± 1.41	4.99 ± 4.80
Vd/F (L/Kg)^a^	23.53 ± 8.56	21.07 ± 14.53
Cl/F (L/h/Kg)^a^	12.18 ± 5.65	9.23 ± 6.36
Half life (h)^b^	1.33 [0.90]	1.36 [3.24]

^a^ Data are expressed as the mean ± SD.

^b^ Data are expressed as the median [IQR].

#### 3.2.2 Single and multiple dose comparison

Most pharmacokinetic parameters of the four diterpenoids were generally consistent between single- and multiple-dose administrations. AP1, AP4, and AP6 exhibited comparable AUC_0-4h_ values across both regimens, indicating no apparent accumulation following repeated dosing. In contrast, AP3 showed a slight reduction in both Cmax and AUC following repeated dosing, suggesting that saturable absorption processes may influence steady-state plasma concentrations.

### 3.3 Pharmacodynamics

#### 3.3.1 Time-course analysis and trend of RNA-dependent RNA polymerase (RdRp) viral load and cycle threshold (Ct)

Viral load reduction was evaluated as an exploratory pharmacodynamic marker in this study and was used to describe the temporal trend of viral decline in patients with mild COVID-19 who received standardized *A*. *paniculata* aqueous extract capsules in combination with standard COVID-19 care. Importantly, viral load reduction was not used to infer or attribute antiviral efficacy to the investigational *A. paniculata* aqueous extract capsules.

**FIGURE 4:**
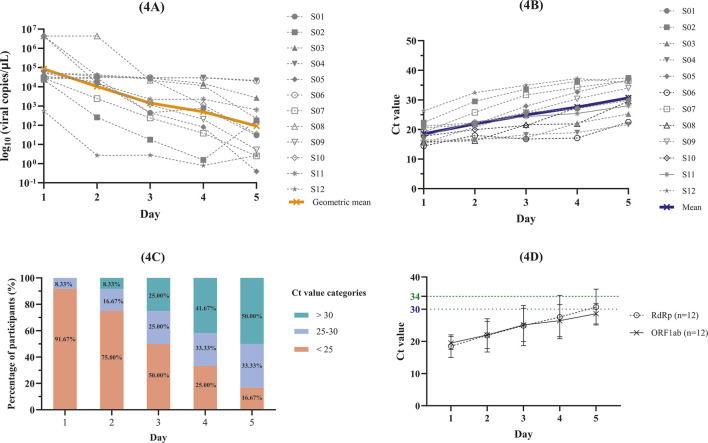
Changes in SARS-CoV-2 viral load and cycle threshold (Ct) values in patients with mild COVID-19 (n = 12). **(A)** RdRp viral load; **(B)** RdRp Ct values; **(C)** RdRp Ct value categories; **(D)** Comparative Ct values of RdRp and ORF1ab.

Baseline RdRp viral loads (day 1) varied across participants (Figure 4A; Table 3), reflecting the natural viral burden typically observed in mild COVID-19 during the early symptomatic phase (within 72 h of symptom onset). By day 2, the geometric mean viral load was decreased from baseline. Maximum viral suppression was observed on day 5, corresponding to a 99.89% reduction in viral burden.

**TABLE 3:** Changes in RdRp viral load in patients with mild COVID-19 during the 5-day period.

Participant	RdRp viral load (copies/μL)					Log_10_ reduction (between day 1 and 5) (−log_10_ copies/μL)	% Reduction (day 1 to day 5)
	Day 1	Day 2	Day 3	Day 4	Day 5		
1	36,075.91	18,077.05	438.59	801.69	29.55	3.09	99.92
2	23,135.56	258.17	17.47	1.55	175.33	2.12	99.24
3	53,580.55	41,508.28	28,969.38	14,647.74	2,638.92	1.31	95.07
4	53,795.48	35,363.79	27,562.61	30,074.15	21,607.30	0.40	59.83
5	26,444.63	28,182.17	438.70	80.45	0.39	4.83	100.00
6	31,074.28	30,436.69	29,976.96	29,066.77	19,680.18	0.20	36.67
7	27,926.22	2,424.48	240.98	38.42	2.51	4.05	99.99
8	4,400,000.00	4,400,000.00	21,691.68	11,488.73	216.00	4.31	100.00
9	52,967.03	14,408.05	1,146.10	205.37	5.21	4.01	99.99
10	4,400,000.00	37,411.22	25,125.89	1,185.48	34.87	5.10	100.00
11	4,400,000.00	15,559.72	2,253.69	2,270.82	635.96	3.84	99.99
12	541.35	2.66	2.76	0.79	2.66	2.31	99.51

Day	GM (copies/μL)	GSD	Range (copies/μL) (min-max)	Log_10_ reduction (−log_10_ copies/μL)	% Reduction
1	8.46 × 10^4^	14.58	5.41 × 10^2^–4.40 × 10^6^	-	-
2	1.03 × 10^4^	30.53	2.66–4.40 × 10^6^	0.92	87.85
3	1.43 × 10^3^	22.74	2.76–3.00 × 10^4^	1.77	98.31
4	5.00 × 10^2^	37.02	0.79–3.01 × 10^4^	2.23	99.41
5	9.21 × 10^1^	35.77	0.39–2.16 × 10^4^	2.96	99.89

Log_10_ reduction calculated as Log_10_ (Day_1_ Geometric Mean/Day_n_ Geometric Mean), % reduction = [1 – (1/10^(log^_10_^reduction)^)] ×100.

Data presented as the Geometric Mean (GM) and the Geometric Standard Deviation (GSD) with range.

In SARS-CoV-2 detection, Ct values function as inverse indicators of viral RNA concentration, with higher Ct values corresponding to lower viral loads and *vice versa*. Baseline RdRp Ct values differed among participants (Figure 4B; Table 4). Ct values increased in all participants over the 5-day dosing period. As shown in Figure 4C, RdRp Ct values were categorized as follows: Ct < 25 indicating high viral load, Ct 25–30 indicating moderate viral load, and Ct > 30 indicating low viral load (Mishra et al., 2022). A shift from higher to lower viral load categories was observed over the 5-day observation period. On day 1, the majority of participants (91.67%) exhibited high viral load, while 8.33% showed moderate viral load, and none demonstrated low viral load. By day 5, most participants (83.33%) were classified within the moderate or low viral load categories, indicating an improvement in virological response.

**TABLE 4:** Changes in RdRp Ct value in patients with mild COVID-19 during the 5-day period.

Participant	Baseline (Day 1)	Day 2	Day 3	Day 4	Day 5	Total increase (day 5- day 1)
S01	21.15	22.24	25.71	27.06	29.82	8.67
S02	22.35	29.50	33.66	36.27	37.46	15.11
S03	15.43	16.81	17.14	22.16	25.22	9.79
S04	16.46	16.44	18.22	18.99	21.66	5.20
S05	20.51	21.77	27.91	32.65	36.78	16.27
S06	14.39	17.97	16.74	17.08	22.55	8.16
S07	19.14	25.78	31.69	34.26	36.53	17.39
S08	15.83	16.18	21.62	21.92	29.56	13.73
S09	15.37	20.80	25.78	30.38	34.02	18.65
S10	17.74	19.97	21.34	27.42	30.65	12.91
S11	17.74	22.66	24.36	25.44	28.04	10.30
S12	26.32	32.45	34.95	37.26	35.87	9.55

Day	Mean Ct value	Standard deviation	Range (min-max)	Ct increase from baseline
1	18.54	3.37	14.39–26.32	-
2	21.88	4.95	16.18–32.45	3.34
3	24.93	5.98	16.74–34.95	6.39
4	27.57	6.44	17.08–37.26	9.04
5	30.68	5.34	21.66–37.46	12.14

In this study, Ct values were assessed using the RdRp target gene, which exhibits higher specificity for SARS-CoV-2, while the ORF1ab gene was used for confirmation due to its higher sensitivity. Both genes are components of SARS-CoV-2 viral RNA commonly used for RT-PCR detection. Analysis demonstrated that Ct values from both target genes were highly comparable (Figure 4D). Therefore, RdRp was used as the primary target gene for viral load assessment and Ct value interpretation throughout the study based on its specificity for SARS-CoV-2.

#### 3.3.2 PK/PD relationship

**FIGURE 5:**
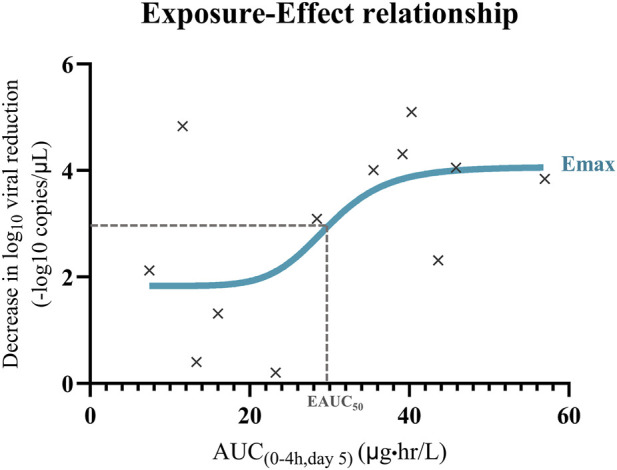
Exploratory PK/PD exposure-effect relationship following oral administration of standardized *A. paniculata* aqueous extract capsules (equivalent to andrographolide 90 mg/day) for 5 consecutive days in combination with standard of care in patients with mild COVID-19 (n = 12).

Viral load was considered as a pharmacodynamic marker to explore potential exposure-response relationship. However, the reductions in viral load cannot be considered as evidence of antiviral efficacy of the *A. paniculata* extract without a placebo-controlled comparison. Andrographolide is the primary bioactive compound among the four major diterpenoids in *A. paniculata* (Songvut et al., 2022; Kongsomros et al., 2024). Accordingly, its AUC was selected as the pharmacokinetic parameter for the PK/PD analysis, and the exposure-effect model was developed to describe the relationship between andrographolide of SARS-CoV-2 (Figure 5).

An exploratory sigmoidal Emax model was fitted to explore the exposure-response relationship between andrographolide exposure (AUC_0-4h_) and log_10_ viral load reduction, with an r^2^ of 0.4. Parameters estimate included EAUC_50_ = 29.80 μg h/L, and Emax = 2.24 log_10_ copies/mL. The Hill coefficient was estimated to be approximately 8 and approached the upper boundary of the constrained parameter range, indicating substantial uncertainty in slope estimation. Goodness-of-fit evaluation using observed versus model-predicted plots and residual diagnostics indicated reasonable agreement with the observed data without apparent systematic bias. However, confidence intervals could not be reliably estimated for Emax, EAUC_50_, and the Hill coefficient due to the small sample size (n = 12), narrow exposure range, and single dose level. Accordingly, the PK/PD analysis should be interpreted as exploratory and hypothesis-generating rather than confirmatory.

### 3.4 Participant demographic and clinical laboratory parameters

Participant baseline demographic characteristics are summarized in Table 5. Participants were predominantly young adults (mean age: 34.08 ± 9.29 years), with two participants classified as middle-aged adults. All participants were vaccinated, and none had significant comorbidities, including cardiovascular disease, diabetes, chronic lung disease, immunosuppression, or other high-risk conditions.

**TABLE 5:** Demographic characteristics of the study participants and clinical laboratory findings at baseline and after oral administration of standardized *A. paniculata* aqueous extract capsules equivalent to 90 mg/day of andrographolide for 5 consecutive days in patients with mild COVID-19.

Demographic data			Baseline (Day 1, n = 12)	End of study (Day 5, n = 12)
Gender, % (n)	Male		16.67 % (n = 2)	16.67 % (n = 2)
	Female		83.33 % (n=10)	83.33 % (n=10)
Age (year)			34.08 ± 9.29	34.08 ± 9.29
Body mass index (kg/m^2^)			21.07 ± 2.19	21.07 ± 2.19
Systolic blood pressure (mmHg)			120.58 ± 9.31	115.08 ± 12.70
Diastolic blood pressure (mmHg)			74.42 ± 7.70	70.83 ± 6.53
Pulse rate (breaths/min)			92.00 ± 13.14	77.00 ± 9.62

Clinical laboratory results		Reference range M (Male), F (Female)	Mean ± SD (min-max)	Mean ± SD (min-max)
White blood cell (×10^3^ µL)^a^		M:3.58-8.15, F: 3.17-8.40	5.26 ± 1.87 (3.24-8.51)	4.74 ± 1.04 (2.95-6.25)
Neutrophil (%)^a^		M: 39.6-67.0, F: 39.7-71.2	61.25 ± 11.59 (41.0-81.6)	52.97 ± 8.82 (39.8-68.0)
Lymphocyte (%)^a^		M: 24.0-48.4, F: 21.9-50.3	26.27 ± 10.84 (9.0-48.5)	38.48 ± 8.74* (22.2-51.9)
Monocyte (%)^a^		M: 4.8-10.1, F: 4.2-9.6	9.33 ± 2.48 (6.5-12.7)	5.08 ± 1.86** (1.0-8.5)
Eosinophil (%)^a^		M: 0.8-5.8, F: 0.6-4.9	2.66 ± 1.98 (0.5-6.5)	2.59 ± 1.48 (1.0-5.8)
Basophil (%)^b^		M: 0.4-1.4, F: 0.2-1.4	0.50 ± 0.19 (0.1-0.8)	0.55 ± 0.56 (0.0-2.0)
Hemoglobin (g/dL)^a^		M: 13.3-16.6, F: 11.0-14.7	13.40 ± 0.65 (12.3-14.4)	12.52 ± 0.83 (11.6-14.1)
Hematocrit (%)^a^		M: 41.3-52.1, F: 35.2-46.7	40.58 ± 1.32 (37.5-42.5)	38.09 ± 1.65 (35.9-41.0)
Red blood cell (×10^6^ µL)^a^		M: 4.29-5.70, F: 3.72-5.06	4.89 ± 0.46 (4.36-5.93)	4.58 ± 0.47 (3.94-5.65)
Platelet (×10^3^ µL)^a^		M: 172-359, F: 167-390	254.75 ± 41.06 (189-310)	263.58 ± 40.87 (183-322)
Fasting blood glucose (mg/dL)^a^		70-110	86.75 ± 13.59 (68-123)	84.09 ± 6.09 (79-101)
Blood urea nitrogen (mg/dL)^a^		6-20	10.08 ± 2.43 (5-13)	10.75 ± 2.42 (6-14)
Creatinine (mg/dL)^a^		M: 0.67-1.17, F: 0.51-0.95	0.77 ± 0.10 (0.64-1.00)	0.69 ± 0.10 (0.60-0.84)
eGFR (mL/min/1.73m^2^)^b^		≤90	104.32 ± 12.67 (79.0-121.0)	112.09 ± 10.72 (97.3-125.0)
Total bilirubin (mg/dL)^b^		<1.2	0.42 ± 0.24 (0.17-0.94)	0.40 ± 0.19 (0.22-0.86)
Albumin (g/dL)^b^		3.5-5.2	4.53 ± 0.21 (4.2-4.8)	4.36 ± 0.31 (3.9-4.9)
Uric acid (mg/dL)^a^		M: 3.4-7.0, F: 2.4-5.7	4.62 ± 1.03 (3.4-6.5)	3.98 ± 0.93 (2.7-5.3)
AST (U/L)^a^		M: <40, F: <32	21.25 ± 4.65 (14-28)	19.50 ± 3.66 (16-26)
ALT (U/L)^b^		M: <41, F: <33	17.08 ± 11.20 (7-49)	15.58 ± 6.93 (5-26)
C-reactive protein (CRP: mg/L)^a^		1-5	13.03 ± 12.15 (1.57-34.86)	2.60 ± 1.82** (0.62-6.16)
IL-6 (pg/mL)^b^		<7	7.23 ± 5.64 (2.5-19.1)	1.93 ± 0.49** <1.5–2.9
Total cholesterol (mg/dL)^a^		<200	185.25 ± 30.37 (147-236)	183.75 ± 34.75 (142-262)
HDL cholesterol (mg/dL)^a^		M: >35, F: >45	66.92 ± 17.60 (36-103)	55.08 ± 13.47 (40-81)
LDL cholesterol (mg/dL)^a^		<130	112.89 ± 23.78 (79.0-156.6)	119.92 ± 31.93 (77.9-185.2)
Triglycerides (mg/dL)^a^		<150	69.67 ± 24.63 (51-140)	87.58 ± 34.83 (63-187)

Abbreviations: eGFR, estimated glomerular filtration rate; AST, aspartate aminotransferase; ALT, alanine aminotransferase; IL-6, interleukin-6; HDL, High-density lipoprotein; LDL, Low-density lipoprotein.

Reference ranges were reported according to the laboratory standard of the Central Laboratory, Chulabhorn Hospital, Bangkok, Thailand.

^a^ Paired T-Test.

^b^ Wilcoxon signed-rank test. Statistical significance was considered at *p* < 0.05 (*) and *p* < 0.01 (**).

The majority of participants were female (83.33%), while male participants accounted for 16.67%. The mean age of baseline participants was 34.08 ± 9.29 years, and the mean body mass index (BMI) was 21.07 ± 2.19 kg/m^2^. For biochemical parameters (Table 5), no deterioration in renal function was observed. Blood urea nitrogen levels remained within normal limits. Liver function markers, including AST and ALT, remained within reference ranges and exhibited no statistically significant differences between baseline and post-treatment values.

Clinical laboratory parameters related to immunomodulatory and anti-inflammatory effects were assessed at baseline and day 5 (Table 5). A significant increase in lymphocyte percentage was observed, with a greater proportion of patients achieving values within the normal range by day 5 compared with baseline, while monocyte percentages were reduced. Concurrently, significant reductions in inflammatory markers (CRP and IL-6) were observed. By day 5, CRP levels normalized in a larger proportion of patients, and IL-6 concentrations returned to within the normal range in all participants.

### 3.5 Safety and adverse events

For adverse events evaluation (Table 6), a total of four adverse events were reported, each occurring in one participant (8.33%), and all were classified as mild in severity. Gastrointestinal disorders included mild burning sensation in the stomach and loose stools, were reported in one participant and deemed possibly related to the study medication. Mild dizziness in one participant was also considered possibly related. No abnormalities in hepatic enzymes (AST or ALT) were observed, and no clinically significant laboratory-based adverse events were detected.

**TABLE 6:** Adverse events following multiple oral administrations of standardized *A. paniculata* aqueous extract capsules equivalent to 90 mg/day of andrographolide for 5 consecutive days in patients with mild COVID-19.

Adverse events	Standardized *A. paniculata* aqueous extract capsules equivalent 90 mg/day of andrographolide (n = 12)		Relation to medication
	n/12 (%) severity		
Gastrointestinal disorders			
Burning stomach	1/12 (8.33%)	Mild	Possible
Loose stool	1/12 (8.33%)	Mild	Possible
Nervous system disorders			
Dizziness	1/12 (8.33%)	Mild	Possible
Investigations			
AST increased	0/12	-	-
ALT increased	0/12	-	-
Others			
Eye discharge	1/12 (8.33%)	Mild	Unlikely

## 4 Discussion

### 4.1 Pharmacokinetics

Following oral administration of standardized *A. paniculata* aqueous extract capsules equivalent to 90 mg/day of andrographolide, the pharmacokinetic profile exhibited rapid absorption and elimination with limited systemic exposure, consistent with the low oral bioavailability previously reported by Panossian et al. (2000) and Songvut et al. (2023b). The systemic exposure did not directly correspond to the capsule content of individual diterpenoids. In particular, AP3 achieved higher plasma exposure than AP1 (Table 2), despite its lower content in the capsule formulation (Table 1). The greater systemic availability of AP3 may reflect its lower Vd/F and clearance compared with AP1. These findings are consistent with the pharmacokinetic behavior observed in earlier investigation conducted in healthy volunteers (Songvut et al., 2023b). The observed differences in systemic exposure are likely related to compound-specific physicochemical properties, including lipophilicity and protein-binding affinity, which influence tissue partitioning and distribution. In particular, the higher Vd/F of AP1 suggests more extensive distribution into peripheral tissues, which may be advantageous for the management of respiratory viral infections, including COVID-19. Preclinical study has demonstrated significant lung accumulation (Bera et al., 2014), supporting its potential activity in the respiratory tract infection.

Among the four diterpenoids, AP4 exhibited the shortest elimination half-life, potentially due to the presence of a C-19 glucoside moiety (Supplementary Figure S2), which may increase hydrophilicity and promote more rapid elimination through enhanced excretion. Although AP4 achieved peak plasma concentrations comparable to those of AP1, its limited overall exposure and shorter residence time suggest a reduced contribution to sustained systemic levels relative to AP1. In contrast, AP6 exhibited the lowest plasma concentrations, likely reflecting lower oral bioavailability or more extensive metabolism and clearance. AP1 exhibited rapid elimination kinetics, with plasma concentrations declining below the limit of quantification within 4 h after administration. This rapid decline in plasma concentrations may reflect extensive tissue distribution and/or metabolic biotransformation, particularly through conjugation pathways including glucuronidation, facilitating elimination (Panossian et al., 2000; Songvut et al., 2023a). Based on first-order elimination kinetics, approximately 97% of a drug is eliminated after 5 half-lives, with near-complete elimination (>99%) occurring after 7 half-lives (Rowland and Tozer, 2011). Considering the short elimination half-life of AP1 (1.21–1.48 h), repeated dosing at 8-h intervals corresponds to approximately 5-7 half-lives and is therefore pharmacokinetically appropriate.

The plasma concentrations of andrographolide observed in our study were considerably lower than the concentrations that have been shown to produce direct antiviral effects in cell-based models (Sa-ngiamsuntorn et al., 2021). This discrepancy may reflect several pharmacokinetic factors, including limited systemic exposure, rapid clearance, and possible renal elimination and metabolic transformation. These processes differ from the hydrolytic or thermal degradation mechanisms described during extract processing (Jaidee et al., 2025).

As reported in previous studies (Panossian et al., 2000; Songvut et al., 2023a), the oral bioavailability of andrographolide varies considerably according to dose, extraction, and formulation characteristics. The reduction in bioavailability observed at higher dose (Panossian et al., 2000) suggests the involvement of nonlinear absorption and oral bioavailability. These pharmacokinetic characteristics may partly explain the relatively low systemic plasma concentrations observed in the present study. Furthermore, variability in bioavailability related to formulation, matrix composition, and type of extract emphasizes the need for product-specific pharmacokinetic characterization.

The study population consisted of relatively young participants (mean age 34.08 ± 9.29 years). It should be noted that the pharmacokinetics of herbal medicines may differ in elderly populations due to age-related changes in drug absorption, distribution, metabolism, and excretion, as well as reduced renal and hepatic function. In addition, disease severity may influence pharmacokinetic behavior and exposure-response relationships.

### 4.2 Pharmacodynamics

A trend toward viral load reduction was observed over the 5-day observation period. Quantification of SARS-CoV-2 RNA targeting the RdRp gene demonstrated an overall decline in viral load (Figure 4A). Correspondingly, an increase in Ct values was observed for the RdRp (Figure 4B) and ORF1ab gene (Figure 4D), indicating a decrease in viral RNA concentration in nasopharyngeal and oropharyngeal specimens. In the present study, a mean viral load reduction of −2.96 log10 copies/mL from baseline to day 5 was observed (Table 3). This represents a pharmacodynamic endpoint used to explore exposure-response relationships but does not constitute evidence of antiviral efficacy.

Higher Ct values are inversely associated with viral infectivity and transmission risk. Previous studies evaluating infectivity over the course of SARS-CoV-2 infection have compared viral growth in cell culture with Ct values obtained from RT-PCR assays. Young et al. (2021) reported that viable SARS-CoV-2 is rarely cultured at Ct values greater than 30 on or after 14 days following symptom onset, indicating low infectivity largely confined to the early phase of the first two weeks. Moreover, Ct values above 34 are generally associated with negligible infectivity, as evidenced by the absence of successful viral isolation in cell culture systems, indicating minimal transmission potential (La Scola et al., 2020). By day 5 of the present study (Figure 4C; Table 4), 50.0% (6/12) of participants achieved Ct values greater than 30, indicating reduced viral shedding and lower transmission potential. In addition, 41.7% (5/12) of participants exceeded the Ct threshold of 34, a level with negligible infectivity (Figure 4D; Table 4).

### 4.3 Pharmacokinetic-pharmacodynamic (PK/PD) relationship

Based on the evidence from previous study (Benjaponpitak et al., 2023), ethanolic extract of *A. paniculata* has been administered to COVID-19 patients at a dosage equivalent to 180 mg/day of andrographolide. However, the ethanolic extracts exhibited lower dissolution (Rangkadilok et al., 2024) and reduced systemic bioavailability (Songvut et al., 2025) compared with aqueous extracts (Songvut et al., 2023b; Rangkadilok et al., 2024). Accordingly, the present study selected an aqueous extract formulation administered at a lower dosage equivalent to 90 mg/day of andrographolide.

The PK/PD analysis focused on andrographolide based on both scientific and regulatory considerations. Andrographolide is the most extensively investigated diterpenoid constituent of *A. paniculata*, with well-documented evidence supporting its biological and pharmacological activities (Sa-ngiamsuntorn et al., 2021; Kongsomros et al., 2024). In addition, AP1 has been designated as the reference compound for standardized *A. paniculata* preparations and product labeling in accordance with Thai FDA requirements. This compound was selected based on established pharmacological relevance and supporting evidence, with defined assay specifications to ensure product consistency and reproducible pharmacological performance. Accordingly, andrographolide was used as the quantitative reference compound for quality control and PK/PD evaluation in this exploratory investigation. However, further investigations examining the pharmacological activities, metabolic bioconversion, and potential contribution to antiviral activity of AP3 are warranted.

Under the current dosing regimen (90 mg/day), the mean AUC_0-4h_ of andrographolide on day 5 (30.12 μg⋅hr/L; Table 2) approached the estimated EAUC_50_ (29.80 μg⋅hr/L, PK/PD model), indicating that the regimen achieved exposures within the modeled exposure-response range. However, dose optimization cannot be determined from this exploratory study conducted at a single dose strength. Due to the limited number of participants, greater variability in viral load reduction was observed at lower AUC levels, indicating limitations of the model in lower exposure range. Further studies with larger sample sizes, particularly at lower AUC values, are needed to strengthen the model.

The absence of a placebo control group constitutes a fundamental limitation of this study, as it precludes definitive causal attribution of the observed outcomes to *A. paniculata* aqueous extract capsules. In mild COVID-19, SARS-CoV-2 viral kinetics follow a characteristic pattern, with peak viral loads occurring around symptom onset, followed by rapid decline through immune-mediated clearance. In vaccinated patients with intact immune function, SARS-CoV-2 infectious viral shedding is reduced, with median clearance times of approximately 5–8 days in breakthrough infections (Garcia-Knight et al., 2022; Maaske et al., 2023). In the present study, participants were enrolled and initiated administration of standardized *A. paniculata* aqueous extract capsules within an early study window, following laboratory confirmation of SARS-CoV-2 infection by RT-PCR and no more than 72 h after symptom onset. Study medication was then continued for 5 consecutive days. The observed reductions in viral load, improvements in inflammatory biomarkers (including decreases in CRP and IL-6), and immunomodulatory changes occurred within this treatment window, which overlaps with the expected period of natural viral clearance in mild COVID-19; therefore, these findings cannot be conclusively attributed to *A. paniculata* treatment effects.

The PK/PD modeling was conducted as an exploratory analysis to generate preliminary hypotheses regarding exposure-response relationships. Given the small sample size (n = 12), single dose level, and narrow exposure range, model parameters should be interpreted as hypothesis-generating rather than definitive. Although the preliminary PK/PD model indicates that the current dosing regimen may approach the estimated EAUC_50_, several important limitations must be acknowledged. The model was based on present data from a small sample size (n = 12) and a single dose level, leading to considerable uncertainty in parameter estimation.

### 4.4 Safety and clinical laboratory parameters

Safety data and clinical laboratory findings indicated that oral administration of standardized *A. paniculata* aqueous extract capsules, at a dosage equivalent to 90 mg/day of andrographolide for 5 consecutive days, was generally well tolerated in patients with mild COVID-19.

The observed reductions in CRP and IL-6, along with an increased lymphocyte percentage and decreased monocyte count, are biologically consistent with the previous reports of andrographolide, including inhibition of NF-κB signaling and suppression of pro-inflammatory cytokine production (Khanal et al., 2021; Wang et al., 2010; Kongsomros et al., 2024). However, without a placebo control group, these changes cannot be definitively attributed to the investigational *A. paniculata* aqueous extract capsules, as mild COVID-19 is characteristically associated with spontaneous normalization of inflammatory markers and immune parameters during natural recovery.

### 4.5 Contribution and future direction

Despite these limitations, this exploratory study provides important preliminary data. The primary scientific contributions of this exploratory study include the first human pharmacokinetic characterization of standardized *A. paniculata* aqueous extract capsules in patients with mild COVID-19, preliminary evidence of an exposure-response relationship, and the demonstration of an acceptable short-term safety profile. Collectively, these findings provide an evidence-based foundation for future placebo-controlled trials designed to further evaluate clinical outcomes in larger patient populations with COVID-19.

### 4.6 Limitations

- The PK/PD modeling represents an exploratory analysis. Exposure-response evaluation using a sigmoidal Emax model indicated a potential association between andrographolide exposure and viral load reduction. However, the small sample size (n = 12), single dose level, and absence of a control group result in substantial uncertainty in parameter estimation. Accordingly, future studies incorporating larger sample sizes, multiple dose levels, and appropriate control groups are required to establish robust and reliable PK/PD relationships.
- The pharmacokinetic analysis was exploratory, with blood sampling limited to a 0–4 h post-dose window. Future studies employing extended sampling windows and more intensive pharmacokinetic profiling will be necessary to fully characterize the terminal elimination phase.
- Despite the higher systemic exposure of AP3 observed in plasma, its pharmacological activities, metabolic bioconversion, and potential contribution to antiviral activity remain to be clarified. Further investigations, including dedicated metabolite-tracking studies and comparative exposure-response analyses, are required to determine whether AP3 is associated with viral load reduction or contributes to other pharmacological activities.
- Clinical efficacy endpoints were not evaluated. This study focused on pharmacokinetic characterization, exploratory exposure-response modeling, and short-term safety assessment. Symptom duration, time to symptom resolution, disease progression, and quality of life were not systematically assessed. Future trials designed to establish therapeutic benefit should consider incorporating validated clinical outcome measures as primary or secondary endpoints.
- The *A. paniculata* aqueous extract capsule was standardized based on andrographolide content, which is widely accepted as a quantitative marker of its pharmacological activity. However, the extract contains additional phytoconstituents, including flavonoids and minor diterpenoids, and their potential contribution to the overall pharmacological activity warrants further investigation.
- The study population was relatively young and consisted exclusively of vaccinated individuals with mild COVID-19 and no significant comorbidities. Therefore, dedicated pharmacokinetic, safety, and efficacy studies are required before clinical use can be recommended in other populations, including elderly individuals (≥65 years), patients with significant comorbidities (such as diabetes, cardiovascular disease, chronic kidney disease, or immunosuppression), other high-risk populations, and patients with moderate or severe COVID-19.

## 5 Conclusion

This study provides the first exploratory pharmacokinetics and clinical PK/PD relationship of standardized *A. paniculata* aqueous extract capsules in patients with mild COVID-19. The four major diterpenoids exhibited rapid absorption and elimination with limited systemic exposure, supporting repeated dosing to maintain adequate exposure over the 5-day dosing period. PK/PD analysis demonstrated a sigmoidal, saturable exposure-response relationship. The pharmacokinetic parameter AUC_0-4h_ on day 5 was comparable to the estimated EAUC_50_ derived from the Emax model. However, dose optimization cannot be determined from this exploratory study conducted at a single dose strength. The dosing regimen (90 mg of andrographolide per day) was well tolerated, with only mild adverse events reported and no evidence of hepatotoxicity or renal toxicity. These findings provide preliminary evidence regarding the pharmacokinetics, dose-exposure characteristics, and short-term tolerability of standardized *A. paniculata* aqueous extract capsules, supporting further investigation in patients with mild COVID-19. Larger randomized, placebo-controlled studies in diverse patient populations, including older adults and patients with comorbidities, are warranted to confirm these observations and to further evaluate clinical outcomes.

## Data Availability

The raw data supporting the conclusions of this article will be made available by the authors, without undue reservation.
