# Supplementary material for: Exploratory pharmacokinetic-pharmacodynamic characterization and safety of standardized Andrographis paniculata aqueous extract capsules in patients with mild COVID-19
Source: Front Pharmacol. 2026 Mar 13;17:1781740. doi: 10.3389/fphar.2026.1781740 (PMC13022710; doi:10.3389/fphar.2026.1781740)
Supplement: Supplementary file 1 [file DataSheet1.pdf]

Supplementary Material

Exploratory pharmacokinetic-pharmacodynamic characterization and safety of standardized *Andrographis paniculata* aqueous extract capsules in patients with mild COVID-19

Phanit Songvut[1,2], Paruspak Payoong[3], Pilailuk Akkapaiboon Okada[4], Noppawan Rittapai[1], Sumitra Suntararuks[1], Jaratluck Akanimanee[1], Nuchanart Rangkadilok[1,2], Duangchit Panomvana[5], Porranee Puranajoti[5], Jutamaad Satayavivad[1,2*]

[1] Laboratory of Pharmacology, Chulabhorn Research Institute, Bangkok, Thailand

[2] Center of Excellence on Environmental Health and Toxicology (EHT), OPS, MHESI, Thailand

[3] Department of Medicine, Chulabhorn Hospital, Chulabhorn Royal Academy, Bangkok, Thailand

[4] National Institute of Health, Department of Medical Sciences, Ministry of Public Health, Nonthaburi, Thailand

[5] Translational Research Unit, Chulabhorn Research Institute, Bangkok, Thailand

*** Correspondence:**

Assoc. Prof. Jutamaad Satayavivad, Ph.D.
Laboratory of Pharmacology, Chulabhorn Research Institute (CRI),
54 Kamphaeng Phet 6 Rd, Lak Si, Bangkok 10210, Thailand
Tel: (66) 2 5538555 ext. 8539/ Fax: (66) 2 5538562
E-mail address: jutamaad@cri.or.th

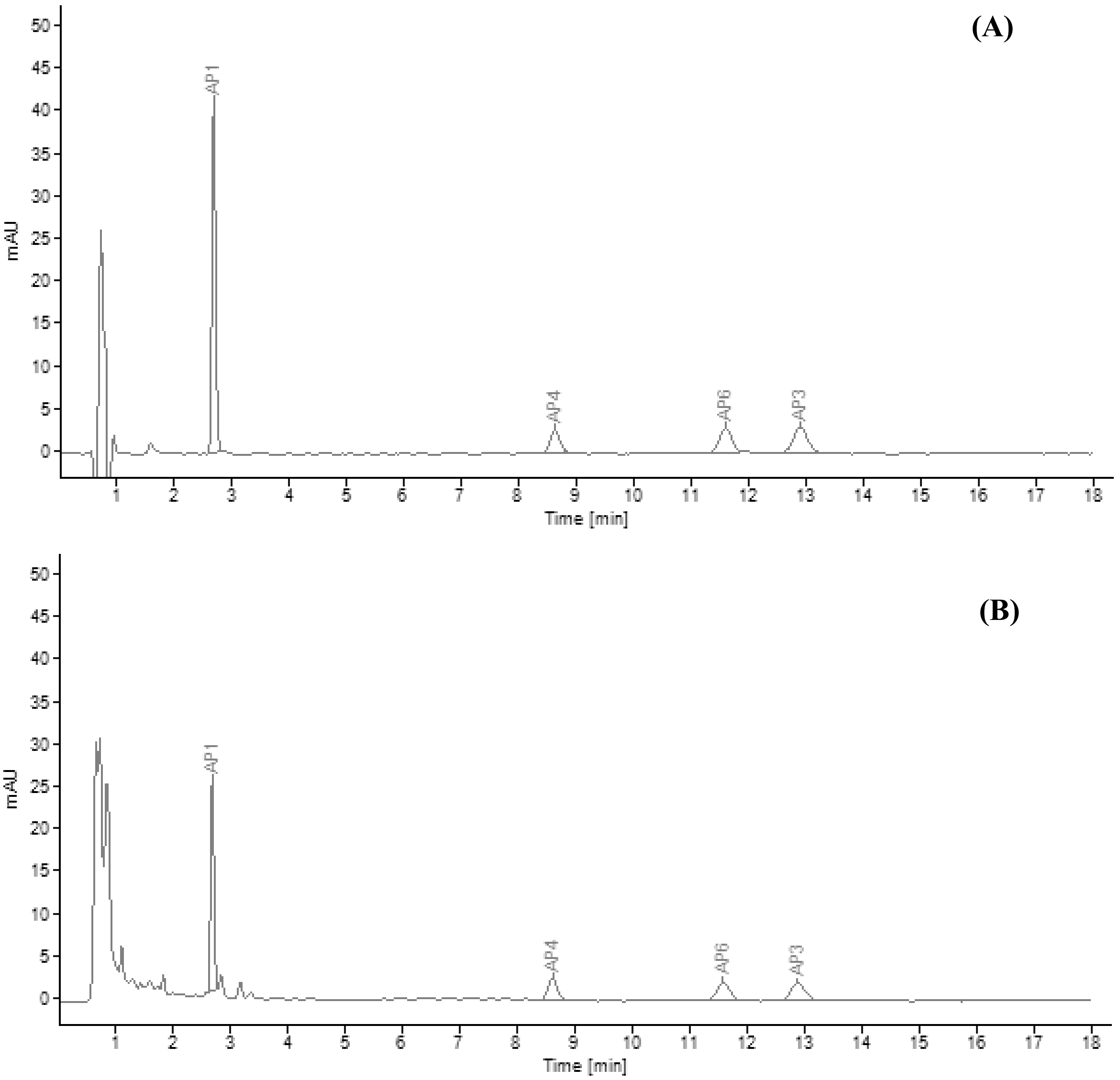

Figure S1. HPLC chromatograms of the four active diterpenoids in *Andrographis paniculata* aqueous extract capsules.

(A) Chromatogram of standard diterpenoids: 25 µg/mL for **AP1**: andrographolide and 5 µg/mL for **AP3**: 14-deoxy-11,12-didehydroandrographolide; **AP4**: neoandrographolide; and **AP6**: 14-deoxyandrographolide

(B) Chromatogram of the four active diterpenoids in the capsule

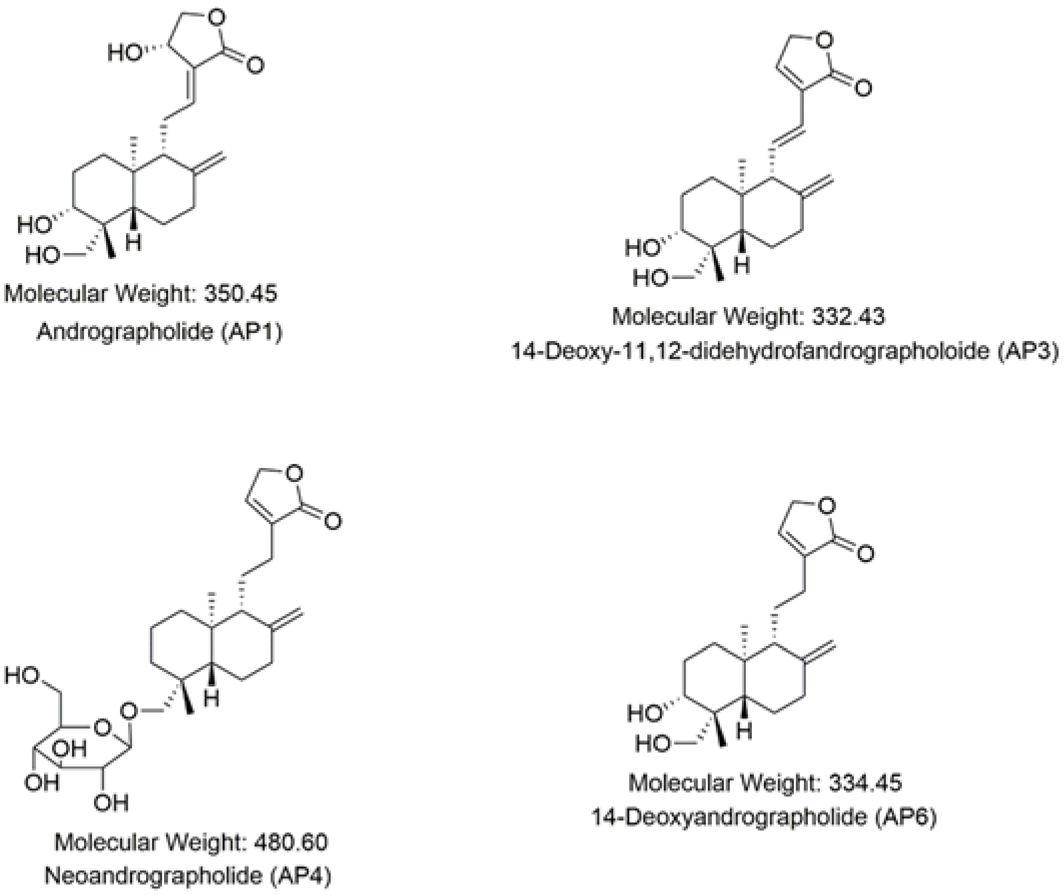

Figure S2. Chemical structures and molecular weights of

AP1: andrographolide;

AP3: 14-deoxy-11,12-didehydroandrographolide;

AP4: neoandrographolide;

AP6: 14-deoxyandrographolide

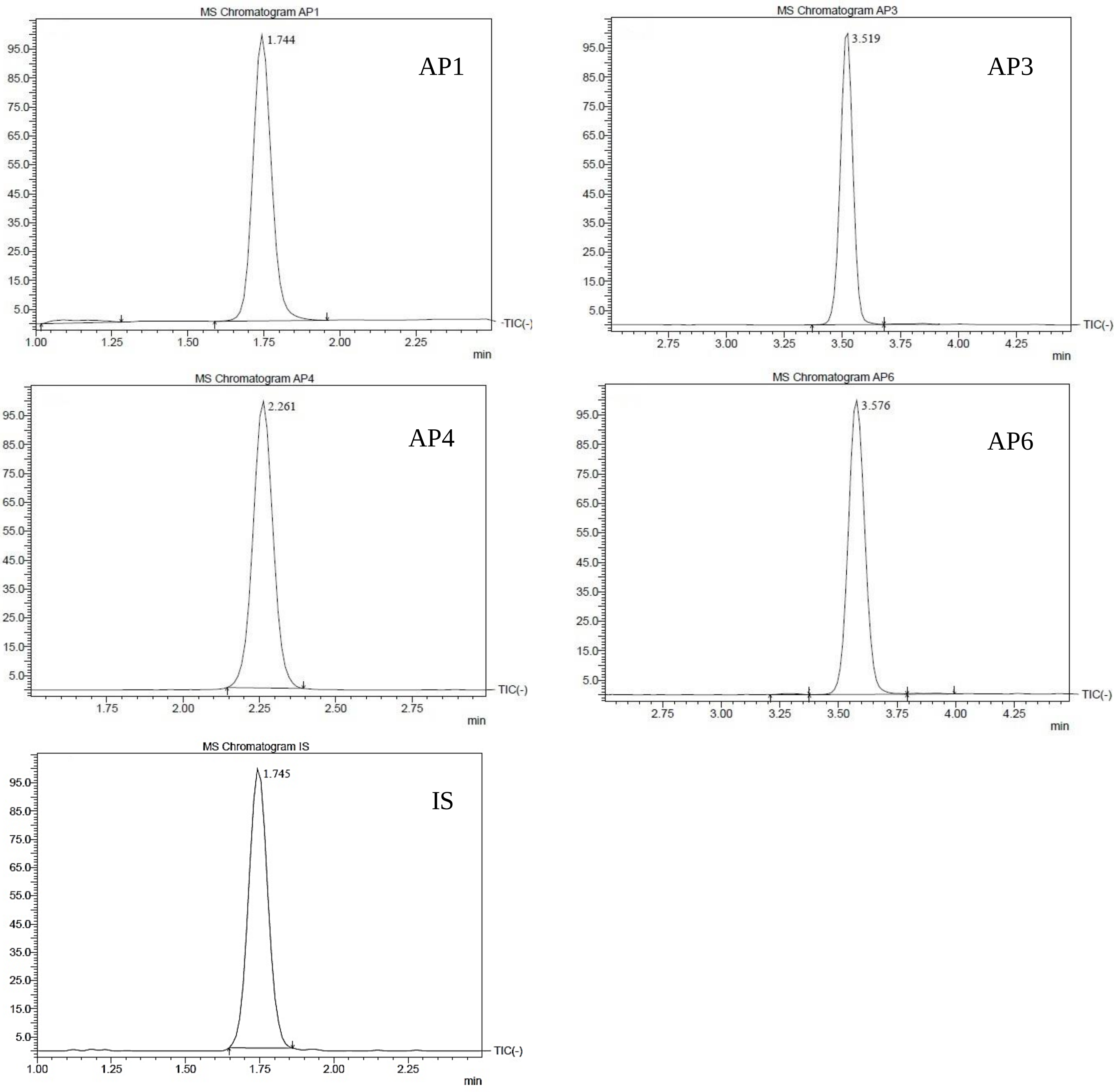

Figure S3. MS chromatogram and retention time of **AP1**: andrographolide; **AP3**: 14-deoxy-11,12-didehydroandrographolide; **AP4**: neoandrographolide; **AP6**: 14-deoxyandrographolide; and Digoxin **(IS)**
